# Primitive Resections of Shoulder and Secondary Myoplastic Operations

**Published:** 1918-12

**Authors:** 


**Primitive Resections of the Shoulder and Secondary Myoplastic Operations.** By M. LETARGET. *Bulletin de la Société de Chirurgie de Paris.* May 14, 1918.

M. Letarget insists on the necessity of sparing carefully the muscular insertions, and limiting the exeresis of the tissues as strictly as possible, while operating on wounded shoulders. He has operated five cases of severe wounds in the shoulder, and practised secondary myoplastic operations.

One of the observations he relates is transcribed here :

G... wounded on July 19; operated on July 21. Deltoid wound, about an inch wide; first dressing with Vincent's powder. X-ray showed a comminutive fracture of the head of the humerus, with fissures extending to one-third of the diaphysis.

Operation : anterior incision ; removal of the fragments of bones and splinters ; cleansing of the bruised soft parts ; extraction of the projectile. Plastered apparatus for two months ; early mobilization and massages.

Nov. 1st : Myoplastic operation. The anterior deltoid bundles are sutured to the superior and external fibres of the large pectoral. This increases the motility of the shoulder. The diaphyseal extremity is just facing the glenoid cavity. The patient can carry his hand to his mouth, but the external and internal rotations are limited.

The other cases are similar to this one. In all of them the suture of the deltoid and the large pectoral has been practised and has improved the movements of the joint.

